# Mechanical deformation of elastomer medical devices can enable microbial surface colonization

**DOI:** 10.1038/s41598-023-34217-5

**Published:** 2023-05-11

**Authors:** Desmond van den Berg, Dalal Asker, Tarek S. Awad, Nicolas Lavielle, Benjamin D. Hatton

**Affiliations:** 1 Department of Materials Science and Engineering, University of Toronto, Toronto, Canada; 2 Institute of Biomedical Engineering, University of Toronto, Toronto, Canada; 3 Food Science & Technology Department, Alexandria University, Alexandria, Egypt; 4 Physique et Mécanique des Milieux Hétérogènes, CNRS, ESPCI, PSL Research University, Sorbonne Université, Sorbonne Paris Cité, 75005 Paris, France

**Keywords:** Bacteria, Biofilms, Microbiology, Materials science, Biomaterials, Soft materials, Disease prevention

## Abstract

Elastomers such as silicone are common in medical devices (catheters, prosthetic implants, endoscopes), but they remain prone to microbial colonization and biofilm infections. For the first time, our work shows that rates of microbial surface attachment to polydimethylsiloxane (PDMS) silicone can be significantly affected by mechanical deformation. For a section of bent commercial catheter tubing, bacteria (*P. aeruginosa*) show a strong preference for the ‘convex’ side compared to the ‘concave’ side, by a factor of 4.2. Further testing of cast PDMS materials in bending only showed a significant difference for samples that were manually wiped (damaged) beforehand (1.75 × 10^4^ and 6.02 × 10^3^ cells/mm^2^ on the convex and concave sides, respectively). We demonstrate that surface microcracks in elastomers are opened under tensile stress (convex bending) to become ‘activated’ as sites for microbial colonization. This work demonstrates that the high elastic limit of elastomers enables these microcracks to reversibly open and close, as ‘dynamic defects’. Commercial catheters have relatively high surface roughness inherent to manufacturing, but we show that even manual wiping of newly-cast PDMS is sufficient to generate surface microcracks. We consider the implication for medical devices that feature sustained, surgical, or cyclic deformation, in which localized tensile conditions may expose these surface defects to opportunistic microbes. As a result, our work showcases serious potential problems in the widespread usage and development of elastomers in medical devices.

## Introduction

Elastomers such as silicones, polyurethanes and polyvinylchloride (PVC) were first used in medical devices in the 1950s and are now in widespread use. Examples include polydimethylsiloxane (PDMS) urinary catheters, polyurethane PICC catheters, endoscope sheaths, and a wide range of reconstructive plastic surgery products, such as silicone prosthetic breast or facial implants^1,2^. Recently silicones have been the basis for a new generation of artificial heart and prosthetic heart valves as silicones have low thrombogenicity, good chemical stability and versatile manufacturability^3^. In addition to implanted devices, elastomers are common in extracorporeal medical devices such as pumps and tubing in dialysis systems.

Despite their widespread use, the microbial colonization of elastomeric devices, and subsequent development to biofilm-based infections, remain a persistent problem both for implanted and reusable devices. Device associated infections are responsible for 50–70% of the nearly 2 million healthcare-associated infections (HAIs) in the US^4,5^. HAIs significantly increase adverse health risks, length of hospital stays for patients and treatment costs. The majority of device-associated infections are a result of bacterial colonization on catheters, including central-line associated bloodstream infections (CLABSI), catheter-associated urinary tract infections (CAUTI), and ventilator-associated pneumonia (VAP)^6,7^. Of the more than 5 million central line catheters inserted per year in the US, 3–5% of these patients suffered from CLABSIs, increasing treatment costs significantly^8^. A recent comparative analysis of patients requiring intravenous catheterization showed infection caused an average of 2 additional days hospital stay^9^.

Device-associated infections begin with the initial colonization of the surface by microbial pathogens and subsequent development into a biofilm^10–12^. Cells in a biofilm produce extracellular polymeric substances, which protect them from disinfectants, antibiotics and host defense mechanisms and as a result biofilms are persistent and difficult to eradicate^5,13–19^. Various gram-positive bacteria (*Enterococcus faecalis*, *Staphylococcus aureus, S. epidermidis*), gram-negative bacteria (*Escherichia coli*, *Klebsiella pneumoniae*, *Proteus mirabilis*, *Pseudomonas aeruginosa*), and fungi (*Candida albicans*) are commonly isolated from explanted medical devices^12^. These pathogens are known to develop multidrug resistance and once they form biofilms the use of systemic, broad-spectrum antibiotics is often ineffective. If infected, the removal and replacement of device is often a necessity, which can be a highly traumatic and medically risky option with a high likelihood for re-infection^12^.

The material factors that determine when and *where* these bacterial colonies initially attach on devices are not well understood. Certainly, there are known risks (probabilities) of contamination events, such as central venous catheters (CVC) picking up flora from skin during insertion or blood-borne pathogens colonizing implant surfaces (in the ‘race to the surface’)^20,21^. However, device-associated infection events remain difficult to predict.

Thus, we aim to better understand the initial stages of microbial surface colonization. In this work we uncover an entirely new mechanism for microbial colonization associated with surface defects and the mechanical deformation of elastomer biomaterials.

**Figure 1:**
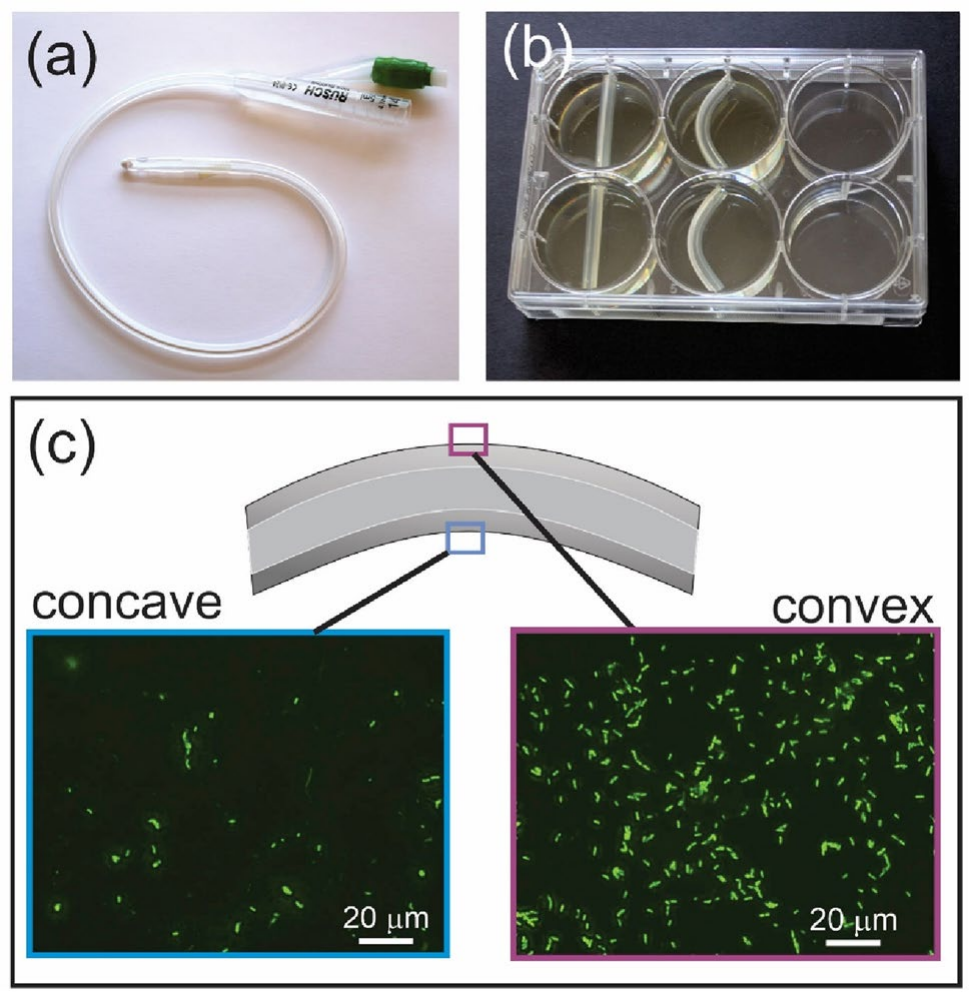
Bending deformation of elastomer devices influences bacterial colonization. (**a**) Commercial silicone Foley urinary catheter (Rusch, OD 4.7 mm, 5 mL); (**b**) Culture of *P. aeruginosa* (25 °C, static culture, LB media) with catheter sections in a straight (unbent) or bent condition; (**c**) Representative fluorescence microscopy images of *P. aeruginosa* (Sytox-stained) on the convex and concave outer surfaces of the catheter tubing, after 4 h growth, showing significantly higher growth on the convex side.

We begin with a preliminary observation that bending deformation of elastomer devices influences bacterial colonization. Sections of a commercial silicone Foley catheter (Rusch OD 4.7 mm, 5 mL) (Fig. 1a) were cut and exposed to a *P. aeruginosa* (PAO1) culture within the wells of a standard 6-well plate (Fig. 1b). The catheter sections were either kept straight (unbent), or were cut slightly long, to cause them to bend within the wells (mid-point displacement of 4 mm, and 1.9 cm radius of curvature). The samples (n = 5) were suspended at the mid-point of wells, so that cells had free access to all surfaces. After 4 h (25 °C), the samples were fixed with glutaraldehyde (GDA) and Tween-20 and stained with Sytox green (30 min).

In fluorescence imaging (Fig. 1c), the bent sections of catheter tubing showed a significantly higher density of cells on the convex (tensile) side (2.11 ± 0.19 × 10^4^ cells/mm^2^), compared to the concave (compressive) side (4.94 ± 0.3 × 10^3^ cells/mm^2^) by a factor of about 4.2. The unbent samples showed a uniform cell density throughout (1.35 ± 0.47 × 10^4^ and 1.39 ± 0.48 × 10^4^ cells/mm^2^ on sides A and B, respectively).

The question is why are the bacteria 4 times more likely to colonize the *convex* surface of these bent catheters? A correlation between bacterial surface attachment and mechanical bending has not been reported before. There are many surface factors that affect rates of microbial surface attachment, including material properties (surface chemistry, charge, roughness and hydrophobicity)^22–25^ and hydrodynamic shear^26^. However, in our case, both sides of the tubing have identical material properties. It is also reported that bacteria can sense mechanical stress^27^. In our case, the cells colonize these tensile or compressed surfaces *after* the tubing is bent, and therefore the cells themselves do not experience strain. If we consider any effects of structural curvature, we can note the radius of bending (50 mm) is orders of magnitude larger than the cell size, so it is unlikely cells ‘notice’ any local curvature differences.

We suggest the most relevant factor explaining the results of Fig. 1 is the due to changes in local surface roughness (R_a_) and topography in elastic bending. Generally it is well-known found that increases in surface roughness at the nano- and microscale cause increased rates of microbial colonization, for a wide range of materials and microbes^25,28–35^. For example, the adhesion rate of *P. aeruginosa* on rough (R_a_ = 0.9 µm) stainless steel surface can be 10^2^ times higher compared to an electropolished (R_a_ = 0.1 µm) steel^36^. Previously, we have also shown a ~ 1 log CFU cm^−2^ reduction in *P. aeruginosa* on mirror finished (R_a_ = 0.09 µm) compared to standard roughness (R_a_ = 0.18 µm) stainless steel^37^. Many studies have noted a strong preferential attachment and alignment to topographical surface defects such as grooves or scratches for various bacterial strains^38–42^. While the strong preference for microbial alignment to surface defects is not well understood, it appears that defects increase cell-surface contact area, protect the cells from shear forces, and greatly enhance the binding potential and surface adhesion^43,44^. Research shows that the initial adhesion sites on rough substrates occurs in pits, cracks, and defect sites^45,46^. Studies in bacterial-probe AFM have also shown that the retention forces are higher for bacteria attached in defect sites on rougher substrates compared to smooth controls^43^.

Elastomer catheters and medical devices formed by extrusion and injection molding typically have an average surface roughness (R_a_) in the range of 50 to 500 nm^47^, with surface defects in the form of grooves or striations formed by extruding conditions common^47–49^. Baldassari et al. in 1994 were among the first to point out that surface defects (grooves, scratches) are preferential sites for bacterial colonization of medical devices^50^. As an example, Buijsssen et al. found that lower silicone roughness reduced both bacterial and yeast fouling rates^49^.

Here we suggest that even when silicone elastomers do show a low surface roughness (R_a_ < 0.5 µm), and appear ostensibly smooth to the eye (high surface reflectance), ‘dynamic’ microcracks are present, which are mechanically opened and closed in elastic bending. Specifically, a certain population of microcracks may be opened (exposed), or closed, on the convex (tensile) and concave (compressive) sides of bent elastomers, but otherwise remain effectively hidden from view when undeformed. This mechanism is enabled by the very large reversible, elastic strains that are characteristic of elastomer materials, and their ability to effectively ‘re-seal’ themselves. This mechanism may have significant implications in further understanding device-associated infection for a wide range of elastomer medical devices in widespread use (catheters, heart valves, prostheses). In this work we demonstrate this deformation-associated ‘dynamic’ defect mechanism for PDMS silicone, for both newly-cast (undamaged) and damaged conditions.

## Results

We tested the attachment of *P. aeruginosa* cells on sections of the commercial PDMS catheter (Fig. 1a) and cast PDMS, cured in flat petri dishes (Sylgard 184, Dow Corning). The inherent average roughness (R_a_) of the catheter and cast PDMS were found to be 1.78 ± 0.3 and 0.1 ± 0.02 µm, respectively. We exposed the catheter and cast PDMS samples (0.5 × 0.5 × 5 cm (HxWxL)) to mild surface abrasion to induce surface damage, by wiping repeatedly with lab tissue (Kim-Wipe, Kimberly Clark, 50 uni-directional wipes). As a result, samples were categorized as ‘new’ or ‘wiped’. All samples were rinsed with deionized water, sterilized with ethanol (70%), then placed into 6-well plates and bent with a mid-point deflection of 4 mm (corresponding to a strain of 3%), while control samples were cut slightly shorter to remain in a straight, unbent condition (Fig. 1b). *P. aeruginosa* (PAO1) inoculated into LBNS growth medium (5 mL, 1% PAO1) was then added to each well plate and incubated at room temperature (25 °C).

**Figure 2:**
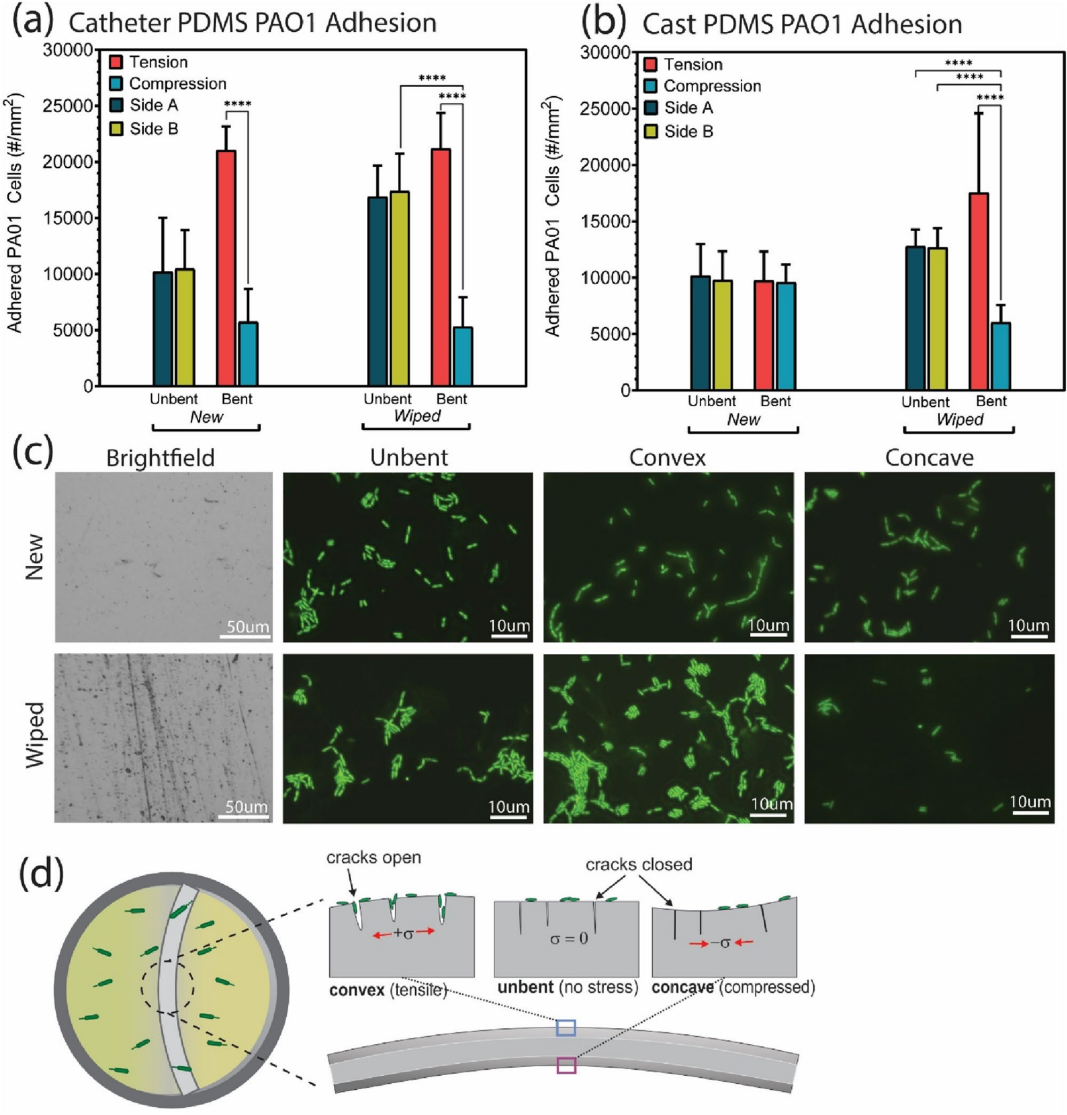
(**a**,**b**) Bacterial colonization of catheter and PDMS surfaces. *P. aeruginosa* (PA01, 25 °C for 4 h) counts by fluorescence image analysis on the unbent, convex and concave surfaces of catheter tubing and cast PDMS, for both ‘new’ and ‘wiped’ samples showing statistically higher attachment on tensile surfaces of damaged silicone (wiped) but not on ‘new’ samples; (**c**) BF and fluorescence imaging of the new and wiped cast PDMS surfaces for the unbent, convex and concave samples. Wiping induced microcracking on the surface, which lead to increased bacterial attachment; (**d**) Schematic of deformation-induced opening of dynamic surface microcracks that enable bacterial colonization. These are opened and exposed in tensile bending, but remain effectively hidden (closed) in unbent and compressed states.

Figure 2a,b shows average cell counts from fluorescence image analysis (cellSens, Olympus BX63) for the catheter and cast PDMS samples, with images for the cast PDMS in Fig. 2c. The catheter PDMS samples showed significantly higher cell density on the convex side of bending in both the ‘new’ and ‘wiped’ conditions, compared to the unbent samples, or concave side. For the cast PDMS there was no measurable effect of bending on cell density for the new (undamaged) samples. However, for the wiped samples of cast PDMS, the bacterial cells again showed a preference for the convex side; 1.75 × 10^4^ cells/mm^2^ (convex), and 6.02 × 10^3^ cells/mm^2^ (concave), a ratio of 2.9. For both catheter and cast PDMS, the wiped samples showed significantly higher cell densities compared to the new (unwiped) samples.

The results of Fig. 2 highlight that the new cast PDMS is ‘immune’ to this effect of tensile deformation in increasing rates of bacterial surface colonization, and that some degree of surface damage is required for this effect to occur. In this case, the surface wiping was enough to generate defects, in the form of microscale scratches, grooves or cracks (as seen in the brightfield images of Fig. 2c). We propose that a population of these surface microcracks, at the scale of microbial cells, are only exposed and ‘visible’ to cells upon tensile deformation (Fig. 2d). Compression of the samples (concave side) appears to generally close these microcracks and make them effectively invisible to the bacteria as attachment sites. Interestingly, the commercial catheter PDMS has sufficient surface roughness, due to extrusion manufacturing, to cause this deformation-induced colonization for both the new and wiped samples (Figs. 1, 2). The newly cast PDMS has very low surface roughness, but basic handling and cleaning procedures (such as wiping), were enough to induce sufficient surface damage to enable the effect of deformation-induced crack opening.

**Figure 3:**
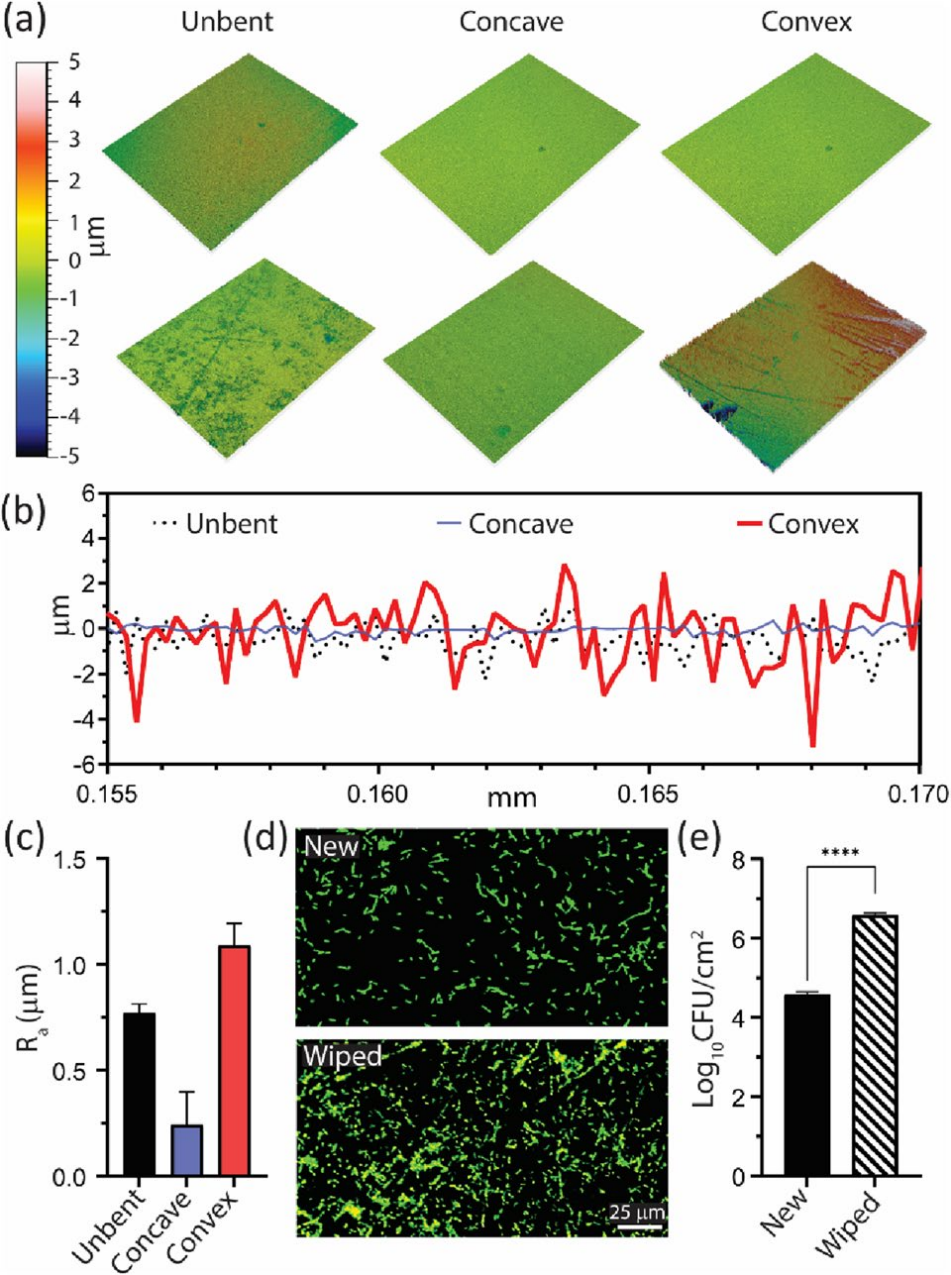
Surface topography and bacterial attachment of new and wiped PDMS exposed to bending. (**a**) Three-dimensional (3D) optical profilometer images showing the surface topography for the unbent, convex and concave samples of new (top row) and wiped (bottom row) sections of cast PDMS; (**b**) Representative surface profiles for ‘wiped’ PDMS; (**c**) Average roughness, Ra, for wiped PDMS; (**d**) Fluorescence microscopy imaging and (**e**) associated cell counts (CFU/cm^2^) for the new and wiped PDMS surfaces (scale bar = 25 μm).

To assess the effect of wiping to elastomer devices, we analyzed the surface topography and measured the average roughness (R_a_) with optical profilometry (Contour GT-3D, Bruker). 3D surface profiles of the cast PDMS (Fig. 3a) showed surface wiping increases surface damage compared to new PDMS. Further, the surface defects generally did effectively ‘disappear’ in compression (concave bending), while increased in both width and depth in tension (convex). The average roughness (R_a_) of the wiped PDMS decreased from 0.75 ± 0.04 μm (unbent) to 0.20 ± 0.02 μm in compression and increased to 1.1 ± 0.1 μm in tension (Fig. 3c). The depth of the ‘opened cracks’ (Fig. 3b) is closely matched to typical bacterial cell sizes, such as *P. aeruginosa* (0.5 μm wide and 2–3 μm long) as seen in the 2D surface profiles for the wiped PDMS (Fig. 3b) where the depth reached values of 3 μm at an average width of 5 μm. After an overnight culture (12 h) of *P. aeruginosa* on newly cast and wiped PDMS, there was a log increase in cell density of 1–1.5 on the wiped sample compared to the smooth control (Fig. 3d,e). The work of Kargar et al. is consistent with these results, which showed a significant increase in cell density if the spacing of defects are wider than the cell width^51^.This further indicates the role of surface topographical defects in providing preferential attachment sites, leading to increased cellular density and biofilm development. The effect of wiping cycles on cast PDMS are further explored in Supplemental Fig. S1. By wiping the surface of cast PDMS between 1 to 100 times, the roughness of cast PDMS increased from a value of 0.1 to 1.25 μm, respectively. Concurrently, increasing wiping cycles showed an increase in the overall attachment of PAO1 to PDMS (2 h incubation of 1% PAO1 in LBNS), highlighting the sensitivity of elastomer materials to surface damage, even when generated through short term wiping contact with a tissue.

Generally, microscale surface defects have not been well-studied or characterized for elastomer biomaterials, and this transient nature of defects and their dependence on dynamic mechanical deformations has never been reported before. Studies aimed at understanding device infections, have noted bacterial colonization on explanted elastomer devices such as catheters and endoscopes on and around surface defects, although these defects are not analyzed in terms of depth or size^50,52–56^. Most relevant to this work is that of Santos et al. who induced damage to endoscope working channels through sequential forcep passes and concluded that this damage increased bacterial colonization due to an increased average roughness (R_a_)^56^. Both the size-dependence of damaged regions and the susceptibility of tensile regions of deformed medical devices to microbial colonization have not been identified before.

**Figure 4:**
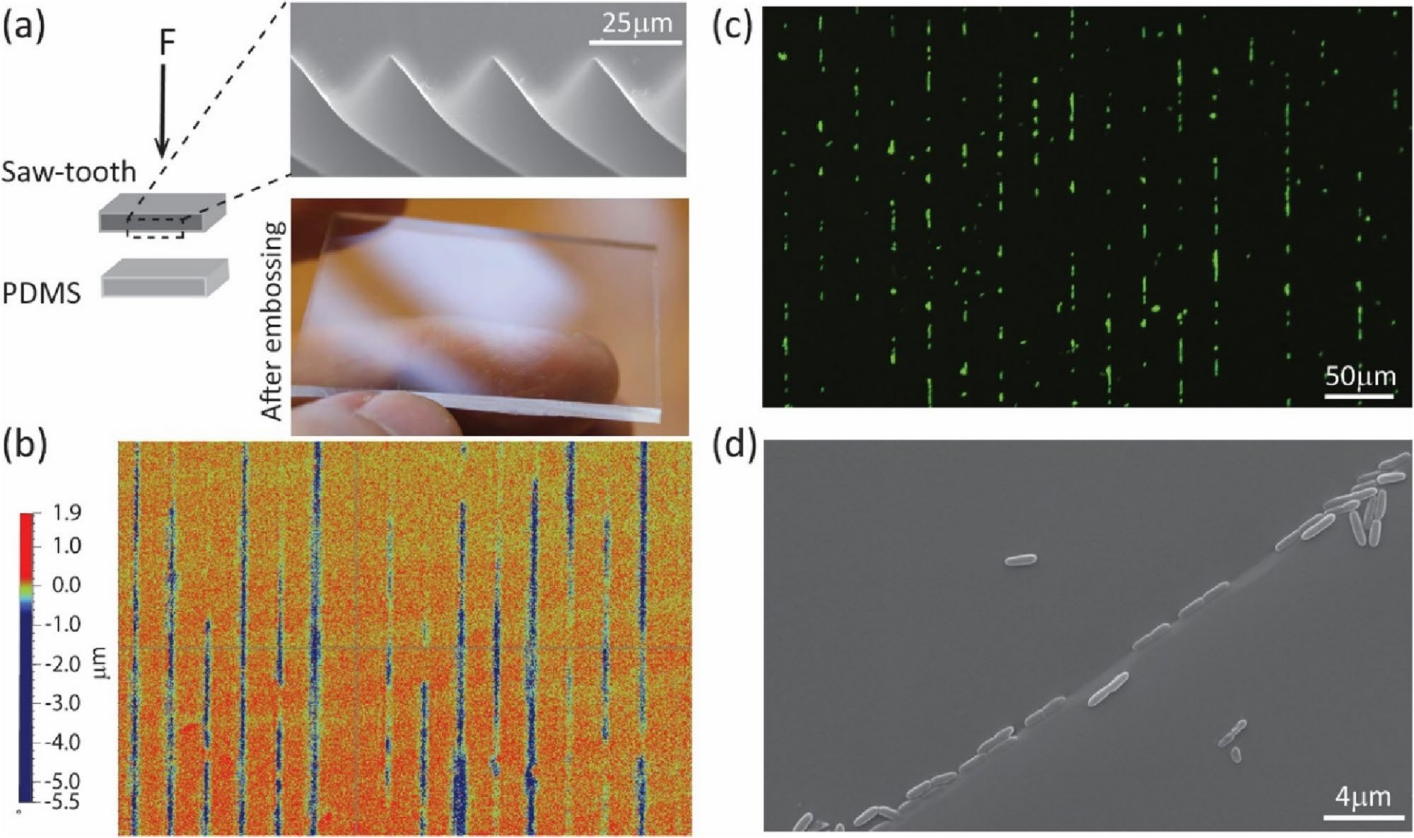
(**a**) Generation of patterned microcracks by ‘press embossing’ a saw-tooth topography (30 µm pitch) against new cast PDMS. The embossed sample appears undamaged to the eye (light reflectance). (**b**) Two-dimensional (2D) Optical profilometry image of the saw-tooth pattern embossed PDMS showing parallel patterned microcracks roughly 4–5 μm deep. (**c**,**d**) Fluorescence and SEM images of *P. aeruginosa* (PA01) attachment on the embossed PDMS samples, showing highly preferential attachment along the patterned microcracks, after 2 h culture (25 °C).

To further test the susceptibility of elastomers to surface damage, we tested the generation of patterned surface cracks in PDMS using systematic, controlled compressive force (EVG 520 semi-automated Hot Embosser). A patterned polystyrene sheet of a ‘saw-tooth’ groove topography (FLEXcon, USA; 30 µm pitch) was pressed against cast PDMS (Fig. 4a). At sufficient force (17 kN over a 5cm^2^ area), optical profilometry imaging (Fig. 4b) of the ‘embossed’ PDMS shows a clear pattern of parallel microcracks, with a maximum depth of 5.5 μm and average depth of 1.4 μm. It is interesting to note that after embossing, the PDMS still macroscopically appears as undamaged (high light reflectance, Fig. 4a). When cultured with *P. aeruginosa* (PAO1) (2 h, 25 °C), the cells showed a very clear preferential attachment to these parallel microcrack defect sites (Fig. 4c). SEM imaging (Fig. 4d) of the PDMS also highlighted the strikingly high degree of alignment of the cells with these patterned surface microcracks. Many cells also appear to be partially trapped within these re-sealed cracks.

## Discussion

Our work raises two unexpected issues about the microbial colonization of elastomer biomaterials. One is that elastomers such as silicones can develop surface damage, in the form of microscale scratches and grooves, through even relatively mild contact such as wiping or localized surface compression, which act as preferential attachment sites for microbes. The second, most importantly, is that some significant population of these defects are reversibly opened and closed through bending deformations, making them ‘dynamic’ as they only become available, and active, under local tensile conditions.

Clinically, this work raises questions about surgical handling procedures for elastomer medical devices, such as wiping, pinching and bending, that may occur in surgery or disinfection (endoscopes), which may be introducing surface microcrack defects. Our work shows that wiping or pressing can introduce highly localized tensile stress states, and enough to cause local fracture or tearing events. The manufacturing of commercial devices itself does appear to already generate such surface defects (as shown in our results for a commercial catheter). The standards for the surface roughness and defect population of medical devices are not well defined. Catheters are subject to approval and acceptance under ISO regulation (ISO 10993, for example), in which the only requirement is that defects cannot be visible to the naked eye or through 2.5 × magnification, whereby micro-scale defects can be easily missed^57,58^.

Surface grooves and microcracks strongly attract initial bacterial attachment (as demonstrated in Fig. 4c), as the first stage of biofilm colony development and device-associated infection, as they provide a protected environment from shear. Surface defects increase the binding affinity through an increase in retention and attachment forces of cells to surfaces^43,45^. There is also evidence that this attachment can be influenced by extracellular appendages (flagella and pilli) to help anchor the planktonic cell^59–61^. In general, patterned regions of the same height or shorter than the length of a flagellum are susceptible to this anchoring, which can be between 5 to 20 μm^34,62^. In this case, the flagellum of the species (a singular polar flagellum for PAO1 of about 5 μm^63^) could easily access the damaged regions, which were about 5 μm wide and 3–5 μm deep. When anchored into these defect sites, the further restriction to flagellar rotation and movement can produce a mechanical signal, which may constitute another example of bacterial mechanosensing.

Our work suggests these dynamic defects may remain effectively hidden or invisible (literally, Fig. 4a) for a device in an undeformed state, but become opened under tension, to enable microbial colonization. Thus, areas of tensile deformation become preferential sites for biofilm initiation. This mechanism of deformation-controlled, dynamic surface defects enabling the initiation of microbial colonization has not been reported before. It appears that these microcracks are able to re-seal again, when relaxed. The mechanism of reversible opening and closing of microcracks is due to the particular mechanical properties of elastomers to allow extremely high, reversible strains without failure; examples include polyurethane (50–100%) and silicones (100–160%)^64,65^. By comparison, the typical maximum strain (elastic limit) for thermoplastics or metals is significantly less; for example, high density polyethylene (HDPE) is just 3%, Ti alloys 1.2–1.5%, and 316L stainless steel 0.1–0.3%. As plastic deformation is initiated beyond the elastic limit, surface cracks cannot open and close reversibly, as they would for elastomers.

We suggest that a wide range of elastomer medical devices may be susceptible to microbial colonization in areas of tensile deformation, beyond just catheter tubing. For example, opened microcracks may effectively ‘carry’ microbes into the body during surgical insertion, protect microbes from disinfection, or provide temporary active sites for opportunistic colonization in cyclic deformation.

**Figure 5:**
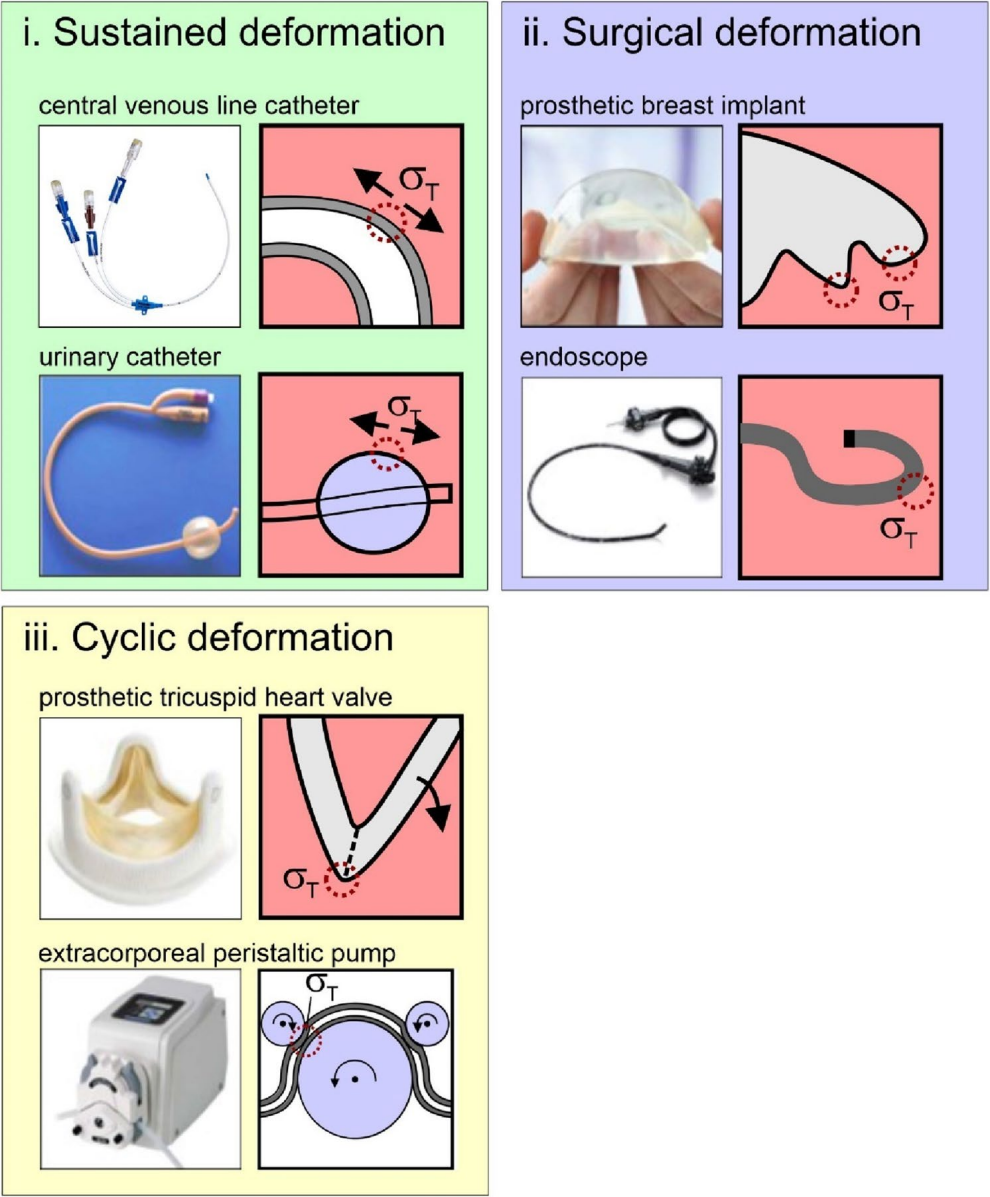
Implanted devices that feature sustained deformation; such as CVC catheter, in bending,^7,20,21^, and urinary catheters (balloon inflation)^69,70^. Medical devices that feature deformation during surgical implantation, such as prosthetic silicone breast implants^71,72^, endoscopes and bronchoscopes^55,73,74^. Other examples include contact lenses, facial implants^75–78^. Finally, devices which feature cyclic deformations, in particular, which can include tricuspid prosthetic heart valves^79–81^, extracorporeal peristaltic pumps (dialysis or ECMO), and prosthetic finger joints^82–86^.

We have considered how medical device deformation may be classified in terms of mechanical deformation, and included examples in each category (Fig. 5). These are devices with; (i) sustained deformation (the device remains deformed while in use); (ii) surgical deformation (the device is deformed during surgical insertion); and (iii) cyclic deformation (periodic deformation). Many devices may experience combinations of these effects.

Examples of sustained deformation include devices such as catheters, shunt or cannula tubing, which are deformed in surgical insertion and remain bent while in use. For example, the deformation of a CVC catheter can exceed 90° to access the central venous bloodstream (and remain in place for weeks), generating local tensile stress. Another example are Foley urinary catheters which feature an expanded balloon (to physically hold in place), and thereby generating significant biaxial tensile stress across the balloon surface. These tensile regions may have a higher probability of biofilm formation, as a result.

Surgically deformed devices can include catheters, tracheostomy tubes, diagnostic devices such as endoscopes or bronchoscopes, and implanted devices such as prosthetic breast implants. Surgical deformation could potentially open microcracks while exposed to skin microflora and contaminated hands, carrying these pathogens into the body. Endoscopes often feature elastomer sheaths, and experience high strains (degree of curvature) within the very high microbial dense areas of the gastrointestinal tract. As discussed below, infectious disease transmission is not uncommon, despite standard disinfection. We suggest areas of tensile deformation, trapping bacteria, may be the reason. Certain implanted devices, such as silicone breast implants, experience high deformations during minimally invasive surgery, achieved by compressing the implants into curved incisions placed around the areola^66,67^. Through the manual and tool mediated compression of these implants to half their initial diameter, large local stresses and abrasive damage can be sustained by the implant^66^. There have been significant clinical problems and controversy associated with silicone breast implants (inflammation, contraction and failure), particularly in the 1990s (resulting in a temporary FDA ban), which was attributed partially to bacterial infection^68^. The root causes could not be traced to the material alone, leading to their re-approval. Based on our experimental results, we speculate that the generation and opening surface microcracks during surgical insertion, contaminated with skin microflora, may have been (and continue to be) a contributing factor to infection complications.

Cyclic deformations occur in devices such as prosthetic heart valves. As an example of biological elastomers, bovine and porcine tissue prosthetic heart valves sustain large strains in use. While native human tissues exhibit a strain at failure of 18–29%, porcine and bovine valves sustain 48–70% and 87–120% strain at failure, respectively^87^. We suggest that this newly-recognized mechanism for deformation-associated microbial colonization may be a contributing factor for Prosthetic valve endocarditis (PVE). Finally, cyclic deformation of elastomers can also occur in extracorporeal devices, such as peristaltic pumps with dialysis or ECMO systems, in the local compression of silicone tubing.

An additional concern is for medical devices which are reused or repurposed such as endoscopes, duodenoscopes and bronchoscopes. Infectious disease transmission between patients remains a concern, despite standard disinfection protocols, for multidrug-resistant bacterial strains of *K. pneumonia, E. coli*, and *Enterococci*^73,74,88–91^. Similarly, endoscopes which are also reprocessed through cleaning steps which include manual wiping, have been shown to become damaged (surface cracks, scratches) and can remain contaminated^53,55,56,92^. Endoscope disinfection often involves coiling the device, putting the inner radii surfaces in compression. While speculative, we hypothesize microbes may be trapped within these closed, re-sealed microcrack environments, and potentially protected from disinfection as a result.

Elastomers and thermoplastic materials are excellent candidates for medical devices from a mechanical and chemical standpoint, as evidenced by their increased prevalence in medical device design. However, device-associated infection remains a persistent problem in healthcare. Our work shows that, ironically, the very high elastic strain limit of these materials may also be contributing to this mechanism for microbial colonization. Our work seeks to define new classifications of medical device deformations for the first time. Each type of deformation may enable these sites of surface damage to be ‘activated’ to increase susceptibility for infection. This work may give us more understanding how and where such infections occur, clinically.

## Methods

### Fabrication of cast PDMS and patterned microcracks

PDMS resin and crosslinker (Dow Sylgard 184) were mixed at a weight ratio of 10:1. PDMS was degassed under vacuum (VWR vacuum oven) at room temperature for 30 min. 25 g of the PDMS was poured into a 100 mm petri dish (VWR) and cured at 60 °C for 24 h. PDMS strips were cut to either 34.8 mm long by 6 mm wide (unbent) or 36.3 mm long by 6 mm wide (bent) to fit into a standard 6 well plate (VWR). Elastomer surface abrasion was done by wiping 50X full 360° rotations with a Kim-wipe tissue in direction parallel to the axis of bending. The patterned surface cracks were generated by ‘press embossing’ (EVG 520 semi-automated Hot Embosser) a saw-tooth topography (30 µm pitch) against newly cast PDMS. All PDMS samples were cleaned by rinsing in DI water and ethanol sequentially, 5X.

### Preparation of bacterial culture

Standard conditions were followed to prepare bacterial cultures. A preculture of *Pseudomonas aeruginosa* PAO1 was prepared by obtaining a single colony from an overnight cultured lysogenic agar media plate incubated at 37 °C overnight. This colony was inoculated into 5 mL of LB and incubated overnight at 37 °C under constant agitation. The bacterial suspension was prepared by adding 1% of the preculture to LB broth with no salt. 10 mL of the bacterial suspension was pipetted into the 6-well plates with the PDMS samples, and incubated for 4 h at 25 °C in static culture. The PDMS samples were rinsed afterwards in 10 mL of 1X PBS buffer three times, and then submerged in a 1% GDA (Sigma Aldrich) saline solution (10 mL) for 20 min. After fixing, the PDMS sections were submerged in a 0.05% Tween-20 (Sigma Aldrich) in saline for 20 min and stained for 30 min using a 50 μL Sytox Green (Life Technologies) in 1X PBS buffer pipetted over each contaminated side of the PDMS. The PDMS was imaged by fluorescence microscopy (Olympus BX63, Japan) using 20X and 50X air objectives, and a GPF filter (λ_ex_/λ_em_ 395/470 nm). Image analysis, image filtering, and cell counts were performed with the Olympus cellSens imaging software.

### Surface characterization

Surface roughness and topography maps were obtained using a non-contact three dimensional Optical profilometer (Bruker Contour GT-K, Tucson, AZ, USA). After calibrating the system scanner, the sample was placed on the microscope stage and the camera was adjusted to focus on the surface microstructure by raising or lowering the z-axis until the appearance then disappearance of two sets of fringes. Measurement was then performed using the vertical scanning interferometry (VSI) mode and focused to determine its upper and lower and upper images using a 20X objective. Results are displayed as 2D contour plot with the X and Y direction cross section plots. Further analysis, using the Bruker Vision 64 Map Premium software, was performed to correct sample tilting and extract surface roughness parameters together with 2D and 3D surface profiles.

## Supplementary Information

Supplementary Figure S1.

## Data Availability

All data will be made available by the authors upon request, please contact D. van den Berg (symen.vandenberg@mail.utoronto.ca).
