# Supplementary material for: Mechanical deformation of elastomer medical devices can enable microbial surface colonization
Source: Sci Rep. 2023 May 11;13:7691. doi: 10.1038/s41598-023-34217-5 (PMC10175502; doi:10.1038/s41598-023-34217-5)
Supplement: Supplementary file 1 — Supplementary Figure S1. [file 41598_2023_34217_MOESM1_ESM.doc]

**Supplemental Information**


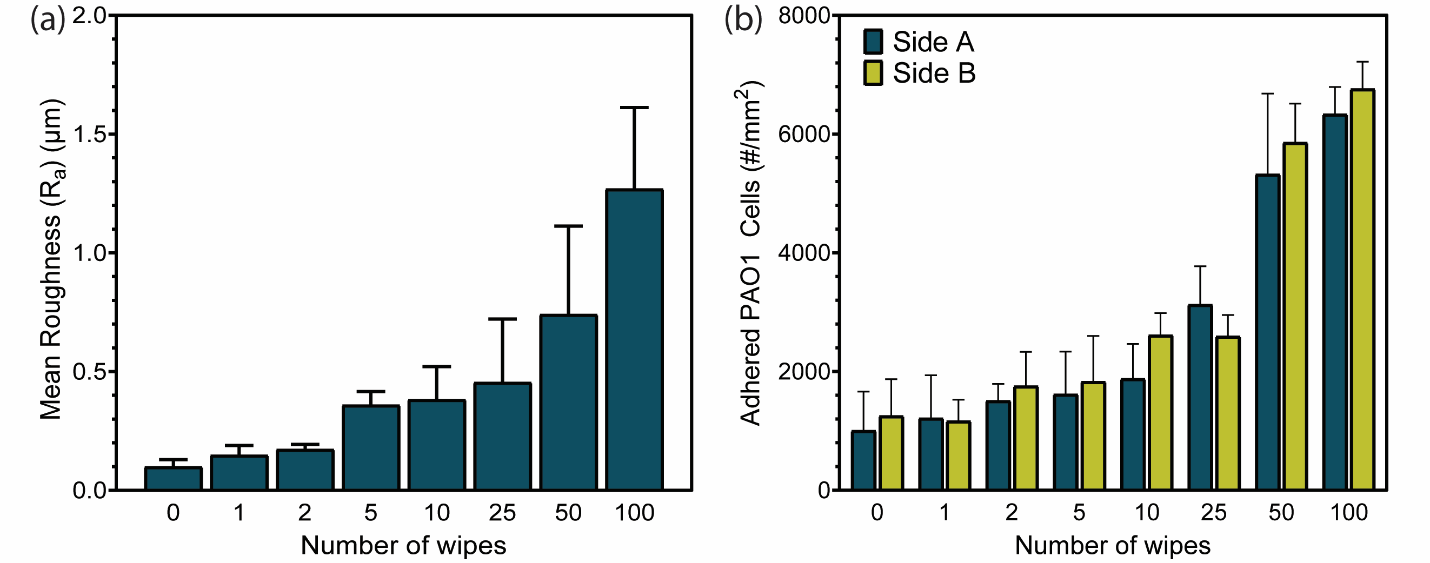
**Do mechanical deformations of elastomer medical devices enable microbial infections?**

**Figure S1.** (a) The effect of increasing wiping cycles on the average mean roughness (R_a_) of cast PDMS. (b) The effect of wiping cycles on the attachment of PAO1 cells to a wiped, unbent section of cast PDMS. Side A and B are equivalent to those shown in prior figures.
